# Complete Genome Sequence of Antibiotic-Producing Bacillus velezensis H208, Isolated from Ginger Rhizosphere in Laifeng County, China

**DOI:** 10.1128/mra.00551-22

**Published:** 2022-12-06

**Authors:** Qi Wang, Yan Gong, Yazhen Yang, Hongtao Hu, Ronghua Zhou

**Affiliations:** a College of Life Sciences, Yangtze University, Jingzhou, China; b Hubei Biopesticide Engineering Research Center, Wuhan, China; c Hubei Hongshan Laboratory, Wuhan, China; SIPBS, University of Strathclyde

## Abstract

The genome of an antibiotic-producing bacterium, Bacillus velezensis H208, was sequenced. Strain H208 was isolated from ginger rhizosphere in Laifeng County, China. The genome consisted of 3,929,792 bp, with a GC content of 46.5%, and contained 3,773 protein-coding genes and 118 noncoding RNA genes.

## ANNOUNCEMENT

Bacillus velezensis was reported as an antagonistic bacterium against various plant diseases (1, 2) and also promotes plant growth (3, 4). It produces multiple antimicrobial secondary metabolites, including antifungal peptides (5), ketone metabolites, and volatile organic compounds (6). B. velezensis H208 was isolated from the soil sticking to ginger roots collected from Zhoujia Village in Laifeng County, Enshi Autonomous Prefecture, Hubei Province, China (109°24′25″E, 29°29′37″N). Ten grams of collected soil and glass beads were added to 99 mL distilled water and shaken at 200 rpm for 30 min at 28°C. The soil suspension was serially diluted 10-fold, and 100-μl aliquots of 10^−3^ to 10^−6^ dilutions were evenly spread on LB agar plates (7). The plates were incubated at 28°C for 24 to 48 h under aerobic conditions until colonies appeared. To obtain pure cultures, the colonies were streaked two or three times on LB agar plates.

Strain H208 was grown in LB liquid medium for 16 h at 28°C, until the optical density at 620 nm reached 0.6 to 0.8. The genomic DNA was subsequently extracted using the Genomic-tip 20/G DNA kit (Qiagen) according to the manufacturer’s instructions. The sequencing library was prepared using the SQK-LSK109 ligation kit 1D (Oxford Nanopore Technologies) without DNA shearing or size selection, sequencing was performed on the Nanopore PromethION platform, and libraries were sequenced with a R9.4.1 flow cell. Sequencing yielded 318,506 raw sequencing reads, with an average length of 5,050 bp. After base calling with Guppy v3.2.6, filtration of sequencing adapters was performed using Porechop v0.2.4 (https://github.com/rrwick/Porechop), and then reads with quality scores of <6 and reads with lengths of <2,000 bp were removed using Filtlong v0.2.1 (https://github.com/rrwick/Filtlong). The remaining reads were submitted to Canu v1.5 (8) for identification and trimming of overlaps (correctedErrorRate=0.12) and genome assembly. Finally, the contig was circularized with Circlator v1.5.5 (9) and rotated based on the *dnaA* gene, and a complete circular chromosome was acquired, consisting of 3,929,792 bp with a GC content of 46.5%. Default parameters were used for all software unless otherwise specified.

The Prokaryotic Genome Annotation Pipeline (PGAP) v6.1 (10) was used to predict a total of 3,891 genes, including 3,773 coding sequences and 118 noncoding RNAs (ncRNAs). The ncRNAs included 86 tRNAs, 27 rRNAs, and 5 other ncRNAs. Moreover, antiSMASH v6.0 (11) analysis of this strain predicted 12 gene clusters for biosynthesis of secondary metabolites, including butirosin (12) and bacillaene (13). Additionally, KEGG annotation of the genes predicted 109 functional pathways, of which the dominant was ABC transporters (10.86%). For further taxonomic identification, the sequenced genome was compared to the closest bacterial genomes deposited in the NCBI database by average nucleotide identity (ANI) analysis using FastANI v1.32 (fragLen=1000, with default parameters for all other settings) (14). Strain H208 was identified to have an ANI value of 98.25% with respect to B. velezensis JS25R (GenBank accession number CP009679.1). The complete genome of B. velezensis H208 would be beneficial for revealing the antagonistic mechanism against plant pathogens and might contribute to the development of new biocontrol agents.

### Data availability.

The raw sequencing reads for B. velezensis H208 have been deposited in the NCBI SRA under accession number SRR21999522, and the genome assembly is available in GenBank under accession number CP097359.
